# Detrimental Impact of Chronic Obstructive Pulmonary Disease in Atrial Fibrillation: New Insights from Umbria Atrial Fibrillation Registry

**DOI:** 10.3390/medicina55070358

**Published:** 2019-07-09

**Authors:** Fabio Angeli, Gianpaolo Reboldi, Monica Trapasso, Adolfo Aita, Giuseppe Ambrosio, Paolo Verdecchia

**Affiliations:** 1 Division of Cardiology and Cardiovascular Pathophysiology, Hospital S. Maria della Misericordia, 06156 Perugia, Italy; 2 Department of Medicine, University of Perugia, 06156 Perugia, Italy; 3 Fondazione Umbra Cuore e Ipertensione-ONLUS and Division of Cardiology, Hospital S. Maria Della Misericordia, 06156 Perugia, Italy

**Keywords:** chronic obstructive pulmonary disease, atrial fibrillation, cardiovascular risk, outcome, prognosis

## Abstract

*Background and objectives:* Chronic obstructive pulmonary disease (COPD) is a leading cause of morbidity and mortality worldwide. Among extra-pulmonary manifestations of COPD, atrial fibrillation (AF) is commonly observed in clinical practice. The coexistence of COPD and AF significantly affects the risk of cardiovascular morbidity and mortality. Nonetheless, the mechanisms explaining the increased risk of vascular events and death associated to the presence of COPD in AF are complex and not completely understood. We analyzed data from an Italian network database to identify markers and mediators of increased vascular risk among subjects with AF and COPD. *Materials and Methods:* Cross-sectional analysis of the Umbria Atrial Fibrillation (Umbria-FA) Registry, a multicenter, observational, prospective on-going registry of patients with non-valvular AF. Of the 2205 patients actually recruited, 2159 had complete clinical data and were included in the analysis. *Results:* the proportion of patients with COPD was 15.6%. COPD patients had a larger proportion of permanent AF when compared to the control group (49.1% vs. 34.6%, *p* < 0.0001) and were more likely to be obese and current smokers. Other cardiovascular risk factors including chronic kidney disease (CKD), peripheral artery disease and subclinical atherosclerosis were more prevalent in COPD patients (all *p* < 0.0001). COPD was also significantly associated with higher prevalence of previous vascular events and a history of anemia (all *p* < 0.0001). The thromboembolic and bleeding risk, as reflected by the CHA_2_DS_2_VASc and HAS-BLED scores, were higher in patients with COPD. Patients with COPD were also more likely to have left ventricular (LV) hypertrophy at standard ECG than individuals forming the cohort without COPD (*p* = 0.018). *Conclusions:* AF patients with COPD have a higher risk of vascular complications than AF patients without this lung disease. Our analysis identified markers and mediators of increased risk that can be easily measured in clinical practice, including LV hypertrophy, CKD, anemia, and atherosclerosis of large arteries.

## 1. Introduction

Chronic obstructive pulmonary disease (COPD) is a major global public health problem, being a leading cause of morbidity and mortality worldwide [1,2]. It is currently rated the fourth most common cause of death and predicted to be the third in the next ten years [2].

Among extra-pulmonary manifestations of COPD, atrial fibrillation (AF) is commonly observed in clinical practice [3,4]. An accumulating body of evidence suggests that COPD is independently associated with this type of arrhythmia [5] and the presence of COPD in AF patients significantly affects outcomes and risk of all-cause mortality [6,7,8,9,10]. In the modern era of anticoagulation therapy, some post-hoc analyses of clinical trials demonstrated that COPD is strongly associated with cardiovascular and non-cardiovascular mortality in AF: data from the Apixaban for Reduction in Stroke and Other Thromboembolic Events in Atrial Fibrillation (ARISTOTLE) trial [9] demonstrated that, after multivariable adjustment for confounders, COPD was associated with a higher risk of all-cause mortality (adjusted hazard ratio [HR]: 1.60, 95% confidence interval [CI]: 1.36 to 1.88, *p* < 0.001) and both cardiovascular and non-cardiovascular mortality. Similarly, the Rivaroxaban Once Daily Oral Direct Factor Xa Inhibition Compared with Vitamin K Antagonism for Prevention of Stroke and Embolism Trial in Atrial Fibrillation (ROCKET AF) [10] showed that COPD was independently associated with higher mortality, suggesting that optimal prevention and treatment of COPD may improve survival.

Nonetheless, the pathophysiological mechanisms underlying the increased risk of vascular events and death associated to the presence of COPD in AF are complex and not completely understood [7]. There is also a paucity of information on risk factors and mediators of vascular events in patients with COPD and AF in most studies [5].

To this purpose, we performed a cross-sectional analysis on a large cohort of AF patients to investigate the interplays between these two conditions. Specifically, we used the unique features of our Italian network database (see methods) to further elucidate and identify markers and mediators of increased vascular risk among subjects with AF and COPD.

## 2. Materials and Methods

### 2.1. Population

We performed a cross-sectional analysis of the Umbria Atrial Fibrillation (Umbria-FA) Registry approved by CEAS-Umbria on date 20 September 2012—Number 1976/12, a multicenter, observational, prospective on-going registry of patients with non-valvular AF (see Appendix A).

Details of this Registry have been reported elsewhere [11]. Enrollment is being performed in 22 Hospitals or out-patient facilities in the setting of the Italian Health System, beginning in January 2013. All patients sign a written informed consent and the study is conducted in accordance with the EU Note for Guidance on Good Clinical Practice CPMP/ECH/135/95 and the Declaration of Helsinki. Each participating centre (see Appendix A) obtained the approval by the Ethics Committees at regional-local levels.

For enrollment, patients must be affected by AF at entry into the Registry or, in case of paroxysmal or persistent AF currently in sinus rhythm, by evidence of AF within one year before entry (standard ECG, ECG-Holter monitoring, or pacemaker diagnostics are also accepted for diagnosis of AF) [11].

Our Italian Network database on AF has the potential to evaluate in detail the key features of AF patients and major gaps in the Guidelines [12,13] implementation in clinical practice, when compared to other registries. Indeed, the initial evaluation of Umbria-FA Registry include a detailed clinical examination, 12-lead electrocardiogram (ECG), laboratory tests and, when feasible, an echocardiographic study.

Based on characteristics of AF episodes, five types of AF are distinguished: first diagnosed, paroxysmal, persistent, long-standing persistent, and permanent AF [12,13]. Standard 12-lead ECG is recorded during brief end-expiratory apnea and left ventricular (LV) hypertrophy at ECG is diagnosed using the BMI-corrected Perugia criterion [14].

The presence of COPD was defined according to documented medical history, as collected by physicians at study site-level. This assessment was performed by any physician during the clinical interview with the patient and by searching through medical records [1]. As for other risk factors, Investigators were asked to follow international guidelines to define COPD [1].

### 2.2. Statistical Analysis

We used STATA 14 (StataCorp, College Station, TX, USA) and R software version 3 (R Foundation for Statistical Computing, Vienna, Austria). Data are presented as mean ± standard deviation (SD) for continuous variables and proportions for categorical variables. Differences in proportions between groups were analyzed using the χ^2^ test. Mean values of variables were compared by independent sample t-test. In 2-tailed tests, *p* values < 0.05 were considered statistically significant.

## 3. Results

**Figure 1:**
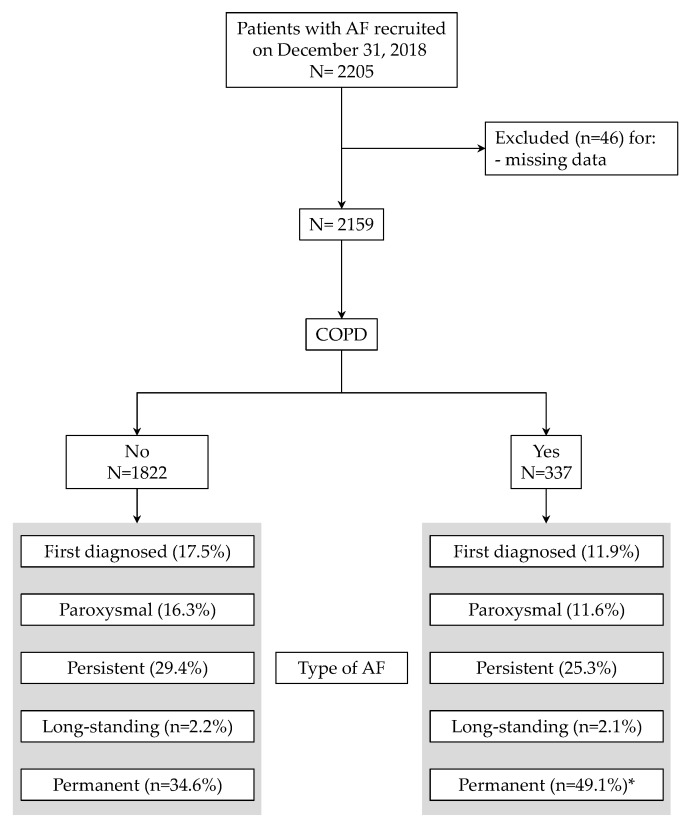
Flow chart of patients through the study. Types of atrial fibrillation are also depicted. * *p* < 0.0001 vs. patients without chronic obstructive pulmonary disease (COPD).

Of the 2205 patients recruited on 31 October 2018 in the Umbria-AF Registry, 2159 had complete clinical data (Figure 1) and were included in the final analysis. The proportion of patients with COPD was 15.6% with a mean age equal to 79.2 ± 8.4 (median: 77). Figure 1 also summarizes the types of AF in the two groups: as depicted, COPD patients had a larger proportion of permanent AF (49.1% vs. 34.6%, *p* < 0.0001). The main characteristics of recruited patients (including risk factors, comorbid conditions and previous vascular events in the two groups) are shown in Table 1.

Patients with COPD were more likely to be obese and current smokers. While hypertension showed a similar prevalence among the two groups, other cardiovascular risk factors (including diabetes and chronic kidney disease (CKD)) were more prevalent in COPD patients (all *p* < 0.0001). Prevalence of patients with established peripheral artery disease (PAD) was 13.9% in the COPD group and 4.7% in the control group (*p* < 0.0001). Subclinical atherosclerosis of large arteries was present in 15% of patients with COPD and 8% in the control group (*p* < 0.0001).

Similar results were obtained for history of previous vascular events: COPD was significantly associated with a higher prevalence of acute coronary syndrome, stroke/transient ischemic attack, and heart failure (HF, all *p* < 0.05). Of note, COPD patients showed an impressive 2.5-fold increase in the frequency of prior HF requiring hospitalization when compared to AF patients without COPD (40.9% vs. 17.4%, *p* < 0.0001). Such different distributions of HF among the two groups translated in lower BP values measured in COPD patients due to LV systolic dysfunction. Indeed, after exclusion of patients with HF from both groups (COPD and non-COPD), none of blood pressure (BP) components showed statistically significant differences between the groups (all *p* ≥ 0.05).

**Figure 2:**
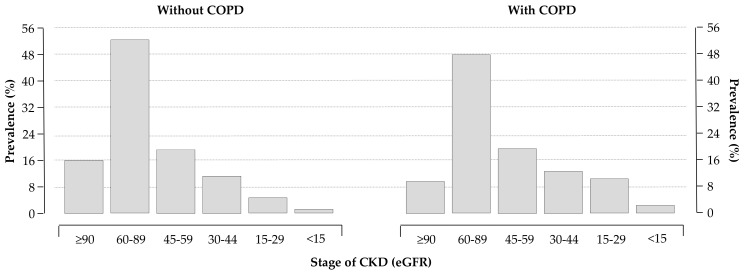
Distribution of chronic kidney disease (CKD) stages in patients without (left panel) and with (right panel) COPD; 28% and 38% of patients without and with COPD had an estimated glomerular filtration rate (eGFR) < 60 mL/min/1.73 m^2^, respectively (*p* < 0.0001).

Routine laboratory data are reported in Table 2. As expected, COPD patients had a lower estimated glomerular filtration rate (eGFR, computed using the CKD-EPI formulas [15]) compared to patients without COPD. Distributions of eGFR classes in the two groups are depicted in Figure 2. 

Of note, COPD influenced the history of previous anemia (24% for COPD patients vs. 14% for patients without COPD, *p* < 0.0001) with mean values of hemoglobin of 12.9 g/dL and 13.5 g/dL for patients with and without COPD at the baseline examination (Table 2), respectively.

**Figure 3:**
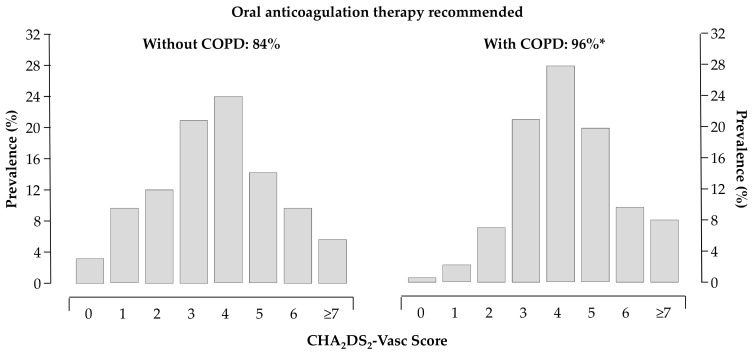
Distribution of CHA_2_DS_2_VASc score in patients without (left panel) and with (right panel) COPD; 84% and 96% of patients without and with COPD had the recommendation to be treated with oral anticoagulants, respectively; * *p* < 0.05 vs. patients without COPD.

The thromboembolic and bleeding risk, as reflected by the CHA_2_DS_2_VASc [16] (4.3 ± 0.09 vs. 3.6 ± 0.04, *p* < 0.0001) and HAS-BLED [17] (1.9 ± 0.06 vs. 1.5 ± 0.02, *p* < 0.0001) scores, were higher in patients with COPD. About 96% of patients with COPD had the recommendation to be treated with oral anticoagulants according to current Guidelines [12,13] (Figure 3).

Among ECG characteristics, 71% of patients with COPD had AF at entry-ECG with a mean heart rate equal to 82 ± 23 b.p.m. In the control group of patients without COPD, 57% showed AF at baseline with a mean heart rate of 77 ± 21 b.p.m. (*p* = 0.0037 vs. patients with COPD). Interestingly, patients with COPD were more likely to have LV hypertrophy at standard ECG than individuals forming the cohort without COPD (45% vs. 38%, *p* = 0.018).

## 4. Discussion

COPD is a major cause of morbidity and mortality and its prevalence is steadily rising resulting in a significant economic and social burden [2]. Although COPD is a predominantly respiratory disease, it has been recently recognized as a systemic disease with significant clinical extra-pulmonary effects and manifestations, leading to a worsening cardiovascular prognosis [7].

A recent study conducted in 7441 patients (mean age 64, 49% women, 92% Caucasian) demonstrated that COPD is an independent predictor of AF onset and progression [3,18]. More specifically, the increased likelihood of AF associated to the presence of COPD remained significant (*p* < 0.0001) after adjusting for several confounders including age, gender, tobacco use, obesity, hypertension, coronary artery disease, HF, diabetes, anemia, cancer, CKD, and rate/rhythm control medications [3].

A wide variety of mechanisms for arrhythmias in COPD seems to exists. In particular, experimental models showed that COPD-related inflammatory responses and hypoxia are implicated in AF development and perpetuation as well [19,20,21]. Furthermore, the coexistence of AF and COPD is a stronger predictor of vascular events than AF only or COPD only [5,7,8,9,10]. Huang et al. reported that the presence of COPD in patients with AF is an independent risk factor for one-year all-cause and cardiovascular mortality [22]; similarly, in the EURObservational Research Programme-Atrial Fibrillation General Registry Pilot Phase (EORP-AF Pilot), COPD was highly prevalent in European AF patients, and was associated with higher rates of cardiovascular death, all-cause death, and the composite outcome of any thromboembolic event/bleeding/cardiovascular death [23]. Furthermore, The Atrial Fibrillation in the Emergency Room (AFTER) Study, derived and validated a complex and a simplified model for the prediction of mortality in the emergency department patients with AF. Of note, both the models that included COPD as a risk variable have been shown to predict mortality after an emergency visit for AF [24].

**Figure 4:**
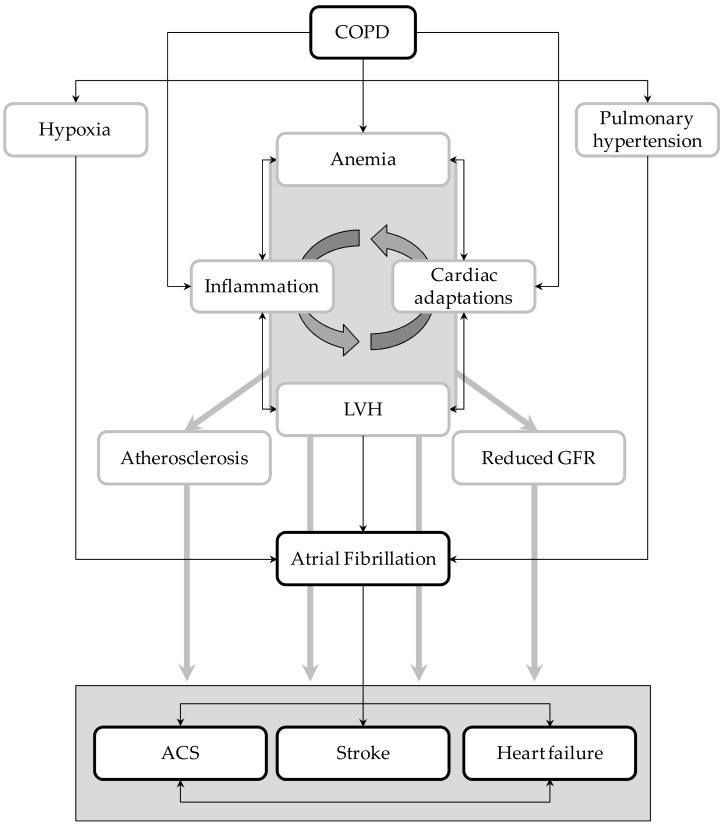
Graphical representation of the potential interplays between atrial fibrillation (AF) and COPD. LVH = left ventricular hypertrophy; ACS = acute coronary syndrome; GFR = glomerular filtration rate.

Nonetheless, the relationships between these two disorders and how they simultaneously act in increasing the risk of vascular events are not completely understood. Taking advantage of the unique features of Umbria-AF registry [11], we tried to elucidate in a large cohort of AF patients the potential mechanisms explaining the increased vascular risk related to COPD. Results of our cross-sectional analysis highlighted the notion that AF patients with COPD have a higher risk of cardio- and cerebro-vascular complications than AF patients without this lung disease. Moreover, our analysis identified potential markers and mediators of high vascular risk that can be easily measured in clinical practice. They include LV hypertrophy, reduced renal function, anemia, and atherosclerosis of large arteries. As summarized below, all these conditions play a central part in the prediction and development of vascular complications (Figure 4).

LV hypertrophy is a powerful and independent predictor of all-cause mortality and major cardiac and cerebrovascular events [25,26]. Experimental evidence is accumulating that several factors. which promote progression of atherosclerosis through plaque growth and destabilization can also induce LV hypertrophy by acting on myocyte and interstitium [25,27]. LV hypertrophy may also be a causative factor for myocardial ischemia and reduced pumping performance and arrhythmias [25,28]. In other words, LV hypertrophy is a marker of cardiovascular risk because it reflects and integrates the long-term level of activity of factors inducing progression of atherosclerosis.

In this context, it is worth mentioning that atherosclerosis of large arteries, as reflected by a wide pulse pressure is significantly related to the risk of major cardiovascular events [29,30,31] and a progressive stiffening of large elastic arteries have been observed in early stages of renal dysfunction [32]. Various studies showed a strong association between the markers of CKD (typically the reduced eGFR) and cardiovascular morbidity and mortality [33,34,35]; more specifically, eGFR is indirectly related to the elevated probability of death and cardiovascular disease [33,34,35]. A recent meta-analysis of clinical studies including 7 million participants reported an increased risk of vascular events and all-cause mortality by 20–30% with a 30% decrease in eGFR [34]. It also suggested that around 20% of vascular events among those over 70 years is attributable to renal dysfunction [34]. Importantly, the complex association of CKD with cardiovascular disease is due to clustering of several cardiovascular risk factors, including “traditional risk factors” (i.e., advanced age, hypertension, diabetes mellitus, and dyslipidemia) and “nontraditional risk factors” that are specific to CKD (i.e., volume overload and anemia). Of note, the presence of anemia may lead to adverse cardiovascular consequences. In the Atherosclerosis Risk in Communities study (ARIC), anemia was associated with cardiovascular outcomes [36], and a significant interaction between anemia and the presence of CKD was documented [37,38]. From a pathophysiological point of view, chronic anemia may increase preload, reduce afterload, and lead to increased cardiac output. In the long term, this may also exacerbate cardiac ischemia as a result of decreased supply or increased demand for oxygen, such as in patients with underlying coronary disease or those with LV hypertrophy, respectively [39].

Taken together, these observations suggest that implications of our results are obvious but clinically relevant at the same time. The elucidation of underlying mechanisms affecting the risk of adverse outcome of AF patients with COPD offers the possibility for better therapeutic options and surveillance strategies. Treatment of AF patients with concomitant COPD should not be the same as those without COPD and physicians need to increase monitoring and early intervention for COPD patients to treat AF.

Our cross-sectional analysis has some limitations. Since white subjects were 99%, caution is needed in extrapolating results to different ethnic groups. Presence or absence of COPD was acknowledged into the electronic case report form reporting the presence/absence of the condition, but with no further details on its severity and lung function testing parameters. The absence of any further details about clinical and severity of COPD is a major limitation. Finally, markers of inflammation are not routinely collected in our Registry. Thus, we are unable to explore the relationships between a pro-inflammatory state and COPD.

## 5. Conclusions

COPD is one of the leading causes of mortality and morbidity worldwide. COPD is an independent risk factor for AF. The coexistence of COPD and AF significantly affects the risk of mortality and vascular events. Thus, it is important to understand the relationship between these two disorders and appropriately manage both these co-morbidities for improved outcomes.

Our cross-sectional analysis produced several findings. First, we highlighted the point that the estimated risk of cardiovascular complications is markedly higher in AF patients with COPD than in those without COPD. Second, we identified markers and mediators of high vascular risk that can be easily measured in clinical practice. Finally, our analysis suggests that specific surveillance strategies and early intervention for COPD patients with AF should be urgently implemented.

## Figures and Tables

**Table 1:** Baseline characteristics of patients included in the analysis.

Variable	Overall (n = 2159)	COPD		*p*
		No (1822)	Yes (n = 3 37)	
Age (years)	75.6 ± 11.2	74.9 ± 11.5	79.2 ± 8.4	<0.0001
Sex (female, %)	44.5	46.3	34.7	0.0001
BMI (Kg/m^2^)	26.8 ± 13.7	26.5 ± 11.9	28.5 ± 20.9	0.0153
Systolic BP (mmHg)	130 ± 18	131 ± 18	127 ± 16	0.0016
Diastolic BP (mmHg)	77 ± 11	77 ± 11	75 ± 11	0.0008
Pulse pressure (mmHg)	53 ± 15	53 ± 15	52 ± 15	0.1960
Heart rate (b.p.m.)	78 ± 22	77 ± 21	82 ± 23	0.0037
Risk factors and comorbid conditions				
Current smoker (%)	6.7	5.8	11.6	<0.0001
Hypertension (%)	80.9	81.2	79.8	0.561
Diabetes (%)	19.7	18.4	26.7	<0.0001
Chronic kidney disease (%) *	29.6	28.1	38.0	<0.0001
Peripheral artery disease (%)	6.1	4.7	13.9	<0.0001
Previous vascular events				
Coronary artery disease (%)	17.8	16.6	24.6	<0.0001
Acute coronary syndrome (%)	14.2	13.5	17.8	0.037
Heart failure (%)	21.1	17.4	40.9	<0.0001
Stroke/Transient ischemic attack (%)	18.1	17.3	22.3	0.029
Pulmonary embolism (%)	1.5	1.5	1.2	0.625

**Legend:** BMI = body mass index; BP = blood pressure; * eGFR < 60 mL/min/1.73m^2^.

**Table 2:** Laboratory data of patients included in the analysis.

Variable	Overall (n = 2159)	COPD		*p*
		No (1822)	Yes (n = 337)	
Haemoglobin (g/dL)	13.4 ± 4.3	13.5 ± 4.6	12.9 ± 2.0	0.0179
Total cholesterol (mg/dL)	170 ± 43	172 ± 43	162 ± 42	0.0013
LDL cholesterol (mg/dL)	100 ± 35	101 ± 35	93 ± 33	0.0024
Serum glucose (mg/dL)	109 ± 34	108 ± 32	115 ± 43	0.0023
Creatinine (mg/dL)	1.08 ± 0.61	1.06 ± 0.58	1.20 ± 0.76	0.0003
BUN (mg/dL)	52 ± 27	50 ± 26	60 ± 34	<0.0001
eGFR (mL/min/1.73m^2^)	67 ± 22	68 ± 22	62 ± 23	0.0001
Uric acid (mg/dL)	6.7 ± 5.3	6.7 ± 5.7	6.8 ± 2.8	0.7657

**Legend:** LDL=low density lipoprotein; BUN=blood urea nitrogen; eGFR=glomerular filtration rate estimated by Cockcroft-Gault equation.

## Appendix A

**Umbria-Atrial fibrillation study group (www.umbriafa.it)**

**Steering Committee**: Giancarlo Agnelli; Giuseppe Ambrosio; Fabio Angeli; Claudio Cavallini; Adriano Murrone; Gianpaolo Reboldi; Paolo Verdecchia (Chairperson); Gianluca Zingarini.

**No-profit Sponsor**: Fondazione Umbra Cuore e Ipertensione—ONLUS.

**Scientific Secretary**: Fabio Angeli.

**Participating Investigators**: Hospital of Ancona and University of Ancona, Department of Cardiology (A. Capucci, F. Guerra, G. Ciliberti); Hospital of Ascoli Piceno, Department of Cardiology (L. Moretti, P. Marchese, F. Gennaro, G. Mazzotta); Hospital of Fabriano, Department of Cardiology (S. Coiro, M. Politano, P. Scipione); Hospital of Amelia, Department of Cardiology (M.L. Suadoni, S. Bergonzini); Hospital of Narni, Department of Medicine (P. Rinaldi); Hospital of Assisi, Department of Medicine (P. Verdecchia, G. Molini, A. Aita); Hospital of Branca, Department of Medicine (S. Radicchia, O. Cazzato); Hospital of Branca, Department of Cardiology (E. Capponi, D. Cosmi, G. Mazzotta); Hospital of Branca, Department of Neurology (D. Giannandrea); Hospital of Città di Castello, Department of Neurology (S. Ricci, M.R. Condurso, L. Greco); Hospital of Città di Castello, Department of Cardiology (A. Murrone, A. Contine, L. Marinacci, K. Mboumi); Hospital of Castiglione del Lago (C. Dembech, N. Sacchi, M. Guerrieri, M. Martinelli); Hospital of Perugia and University of Perugia, Medicina Interna e Vascolare (G. Agnelli, M.G. De Natale, C. Becattini, M.C. Vedovati); Hospital of Perugia and University of Perugia, Cardiologia e Fisiopatologia Cardiovascolare (F. Angeli, F. Scavelli, M. Reccia, G. Giuffrè); Hospital of Perugia and University of Perugia, Medicina Interna (M. Pirro, V. Bianconi, A. Labate); Hospital of Perugia, Struttura Complessa di Cardiologia (C. Cavallini, G.L. Zingarini, F. Notaristefano, C. Riccini); Hospital of Perugia, Struttura Complessa di Pronto Soccorso (P. Groff, V. Mommi); Hospital of Foligno, Struttura Complessa di Cardiologia (G. Bagliani, C. Andreoli, C. Mangialasche); Hospital of Orvieto, Struttura Complessa di Cardiologia (R. Di Cristofaro); USL Umbria 1, Cardiologia Ambulatoriale 1 (M.G. Pinzagli); USL Umbria 2, Cardiologia Ambulatoriale 2 (L. Filippucci, A. Faleburle); USL Umbria 2, Cardiologia Ambulatoriale (S. Repaci), USL Umbria 2, Cardiologia Ambulatoriale (G. Proietti); Hospital Media Valle del Tevere, Struttura Complessa di Medicina (U. Paliani, C. Fuoco, M.G. Conti, A. Cardona, C. Bartolini); Hospital of Terni, Struttura Complessa di Cardiologia (G. Carreras, E. Boschetti, C. Poltronieri, A. Crocetti, G. Tilocca, G. Khoury); Hospital of Terni, Medicina Interna (G. Vaudo, G. Pucci, L. Sanesi, R. Sgariglia, S. Alessio, A. Cerasari, I. Dominioni).
